# Efficient and Selective Photocatalytic Transformation of CO_2_ to CO with Mo_6_ Clusters Supported on Fe-Doped g‑C_3_N_4_

**DOI:** 10.1021/acsaem.5c02019

**Published:** 2025-10-08

**Authors:** Jhon Sebastián Hernández, Marta Feliz

**Affiliations:** 83167 Instituto de Tecnología Química (Universitat Politècnica de València − Agencia Estatal Consejo Superior de Investigaciones Científicas), Avd. de los Naranjos s/n, 46022 Valencia, Spain

**Keywords:** molybdenum clusters, iron, graphitic carbon nitride, CO_2_ photoreduction, nanohybrid material

## Abstract

The carbon dioxide reduction reaction (CO_2_RR) driven by sunlight is expected to become a sustainable way of producing high-value compounds in the future. To achieve this goal, affordable and efficient photocatalysts still need to be discovered. In this work, we show that near-IR luminescent octahedral molybdenum iodido clusters decorated with isonicotinato ligands, (Bu_4_N)_2_[Mo_6_I_8_(O_2_CC_5_H_4_N)_6_] (Mo_6_), once combined with iron-doped carbon nitride (Fe-*g*-C_3_N_4_), provide the Mo_6_/Fe-*g*-C_3_N_4_ nanostructured materials, which are able to photocatalytically produce carbon monoxide (CO) from CO_2_ with high efficiency and selectivity. In a plausible mechanism, the Mo_6_ cluster acts as a photosensitizer, and its pyridine groups interact coordinatively with the embedded iron atoms, thus promoting the electronic conduction to the catalytic iron sites of the nanohybrid. The materials were characterized analytically, texturally, structurally, and spectroscopically using techniques such as inductively coupled plasma (ICP), specific surface area measurements, UV–vis–NIR diffuse reflectance spectra (DRS), powder X-ray diffraction (PXRD), Fourier transform infrared spectroscopy (FTIR), X-ray photoelectron spectroscopy (XPS), field emission scanning electron microscopy (FESEM) coupled with energy-dispersive X-ray spectroscopy (EDS), and photoluminescence. The CO_2_RR studies showed that this association induces a change in the selectivity and a significant increase in CO production compared to that produced separately by the Mo clusters and graphitic precursors. Considering the versatility of this building block strategy for preparing multicomponent hybrid nanomaterials, molybdenum metal clusters are regarded as promising catalysts for creating eco-friendly and cost-effective photocatalysts for the CO_2_RR.

## Graphical abstract

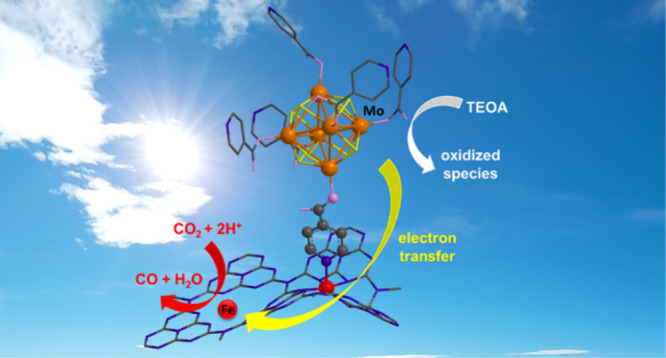

## 1 Introduction

The increasing concentration of CO_2_ in the atmosphere represents one of the major global environmental concerns, significantly contributing to climate change and its devastating consequences. The pursuit of innovative and sustainable approaches to mitigate this issue has become a crucial priority in contemporary scientific research. In this context, new photocatalytic materials have emerged as a promising strategy for CO_2_ removal promoted by sunlight.^1–5^

The development of nanostructured photocatalysts with unique properties and enhanced catalytic performance is currently a great challenge. In fact, the modification of high-surface semiconductors as platforms to incorporate suitable photoactive and catalytically active molecules results in highly efficient hybrid materials with future prospects of sustainable transformations promoted by light in the framework of artificial photosynthesis and sustainable chemistry research. These materials leverage the synergetic effects of their individual components to offer a superior functionality compared to single-component systems.^6,7^

Graphitic carbon nitride (g-C_3_N_4_), a material derived from the family of carbonitride polymers, has captured the scientific community’s attention due to its unique properties, including high stability, abundance, and the ability to absorb visible light.^8,9^ These characteristics and the easy chemical modification of g-C_3_N_4_ with other catalysts and promoters make g-C_3_N_4_ and its composites ideal candidates for catalyzing the conversion of CO_2_, a greenhouse gas, into fuels such as CO as a valuable chemical compound.^1,10^ Graphitic carbon nitride has also shown great potential in other applications such as photocatalytic hydrogen production,^1,8,11,12^ energy conversion and storage,^10^ as well as other photocatalysis processes, such as pollutant removal,^4,5,13,14^ organic synthesis,^15^ and biosensing.^16^ However, pure g-C_3_N_4_ exhibits some problems due to the low visible light utilization, small specific surface, and high rate of charge carrier recombination. The modification through metal doping is a methodology that allows overcoming these disadvantages and modifying the response of photocatalysts to improve the physicochemical properties.^4,5,10,11,16^ This is because metals can act as electron acceptors, facilitating efficient charge carrier separation. Furthermore, this modification can broaden the absorption spectrum of the photocatalyst. This means that the modified catalyst can harness a wider range of light wavelengths, including visible light, which is abundant in sunlight.^2,4,10,16^

As we face increasingly pressing environmental challenges, a thorough understanding and effective application of metal (M)-doped g-C_3_N_4_ (M-*g*-C_3_N_4_) catalysts in the CO_2_RR become critically important in the transition to more sustainable practices and in the search for solutions to mitigate the impact of climate change. However, M-*g*-C_3_N_4_ materials have been primarily used in photodegradation of organic contaminants and water splitting, and the reactivity is dependent on the nature of the dopant.^3,4,15^ Thus, while Fe-*g*-C_3_N_4_ materials have proven to be active for oxidation processes, an effective functionalization enabling their use in reduction processes is less known.^17–20^ To the best of our knowledge, only Hao and co-workers reported a mechanistic study of the CO_2_RR to CO catalyzed by an atomically dispersed Fe on the g-C_3_N_4_ material, with low surface area and catalytic performance (less than 1 μmol g^–1^ h^–1^).^21^

The decoration of carbon nitride materials with other inorganic components by covalent or supramolecular interactions is an alternative strategy to obtain structured derivatives with enhanced properties.^6,7^ Hexamolybdenum halide octahedral clusters emerge as suitable candidates for their incorporation onto carbon nitride surfaces. These nanosized compounds, denoted as [Mo_6_X_8_L_6_]^n^ (n = charge), are robust metal units consisting of internal halide ligands (X) and apical (or terminal) ligands (L) of either organic or inorganic nature.^22^ These clusters show intrinsic electronic and optical properties, with an intense emission in the red and near-infrared (NIR) regions, featuring high quantum yields (Φ_em_ up to 88%) and relatively long lifetimes in the microsecond range (τ_em_ up to ∼300 μs) associated with phosphorescence.^23,24^ The octahedral molybdenum clusters have been shown promising applications in photocatalytic hydrogen evolution reaction,^25^ biomedical applications,^26^ sensors,^27–29^ and photonics.^30,31^ In the field of the CO_2_RR, the incorporation of {Mo_6_Br_8_}^4+^ core clusters on the surface of graphene oxide enhanced the photocatalytic efficiency in the CO_2_RR to methanol in the presence of water,^32^ offering an intriguing strategy worth considering. As far as we know, the interaction of the molybdenum cluster with carbon supports modified with metal dopants, as well as its physicochemical and photocatalytic properties, has not been studied. In this work, Mo_6_/Fe-g-C_3_N_4_ nanostructured hybrids were prepared from Mo_6_ and Fe-g-C_3_N_4_ materials, and the catalytic activity and selectivity were assessed in the CO_2_RR.

## 2 Results and Discussion

### 2.1 Synthesis and Characterization

The Mo_6_/Fe-*g*-C_3_N_4_ nanocomposites were prepared by immobilization of the Mo_6_ cluster compound onto Fe-*g*-C_3_N_4_ supports, with different iron contents. The Mo_6_I_8_ cluster unit bearing isonicotinato ligands is considered a suitable building block for incorporation into Fe-*g*-C_3_N_4_ supports by coordinative interactions with iron atoms (Figure 1a), as demonstrated in similar molecular systems reported by Sokolov et al.^33,34^ The iron-doped graphitic supports were obtained in a two-step synthesis: first, pristine g-C_3_N_4_ was obtained by thermal polycondensation of melamine, followed by a two-step thermal exfoliation to provide a 10-fold increase of the surface area (ca. 118 m^2^·g^–1^).^35–39^ Second, iron­(III) chloride (1, 3, and 7% w/w) reacted with aqueous g-C_3_N_4_ at 90 °C to give Fe-*g*-C_3_N_4_-1, Fe-*g*-C_3_N_4_-3, and Fe-*g*-C_3_N_4_-7, respectively. The specific surface area of the Fe-*g*-C_3_N_4_ materials (114 m^2^·g^–1^ avg., Table S1) did not change substantially with respect to the graphitic precursor, whereas other authors observed an increase in area after metal doping.^13,38^ The preparation of Mo_6_/Fe-*g*-C_3_N_4_ was achieved by mixing the molecular Mo_6_ cluster with the Fe-*g*-C_3_N_4_ support in THF with soft heating (40 °C) overnight. The incorporation of the molecular cluster induces a slight decrease in the surface area (9.5 m^2^·g^–1^ avg., Table S1), which is mainly attributed to pore plugging in the support.^40^ The metal content in the graphitic compounds was determined by ICP spectroscopy (Table S1). The presence of iron was confirmed in Fe-*g*-C_3_N_4_-1, Fe-*g*-C_3_N_4_-3, and Fe-*g*-C_3_N_4_-7 materials, indicating that the method used for metal doping of g-C_3_N_4_ was effective. The molybdenum content in the three corresponding Mo_6_/Fe-*g*-C_3_N_4_ nanomaterials is approximately 0.2% (w/w) in all cases (this value corresponds to roughly 0.8% of the cluster). Furthermore, after the cluster immobilization process, the Fe concentration in the materials remained unchanged. At least five attempts were made using different amounts of cluster (5, 10, 20, 30, and 50 mg) and various reaction times (2, 6, and 24 h) to increase the Mo_6_ content in the materials; however, none were successful. These results suggest that, regardless of the Fe concentration incorporated into the graphitic material, only a fraction of the Fe atoms would be accessible for the coordinative anchoring of Mo_6_ through pyridino groups, in agreement with the model depicted in Figure 1a.

**Figure 1:**
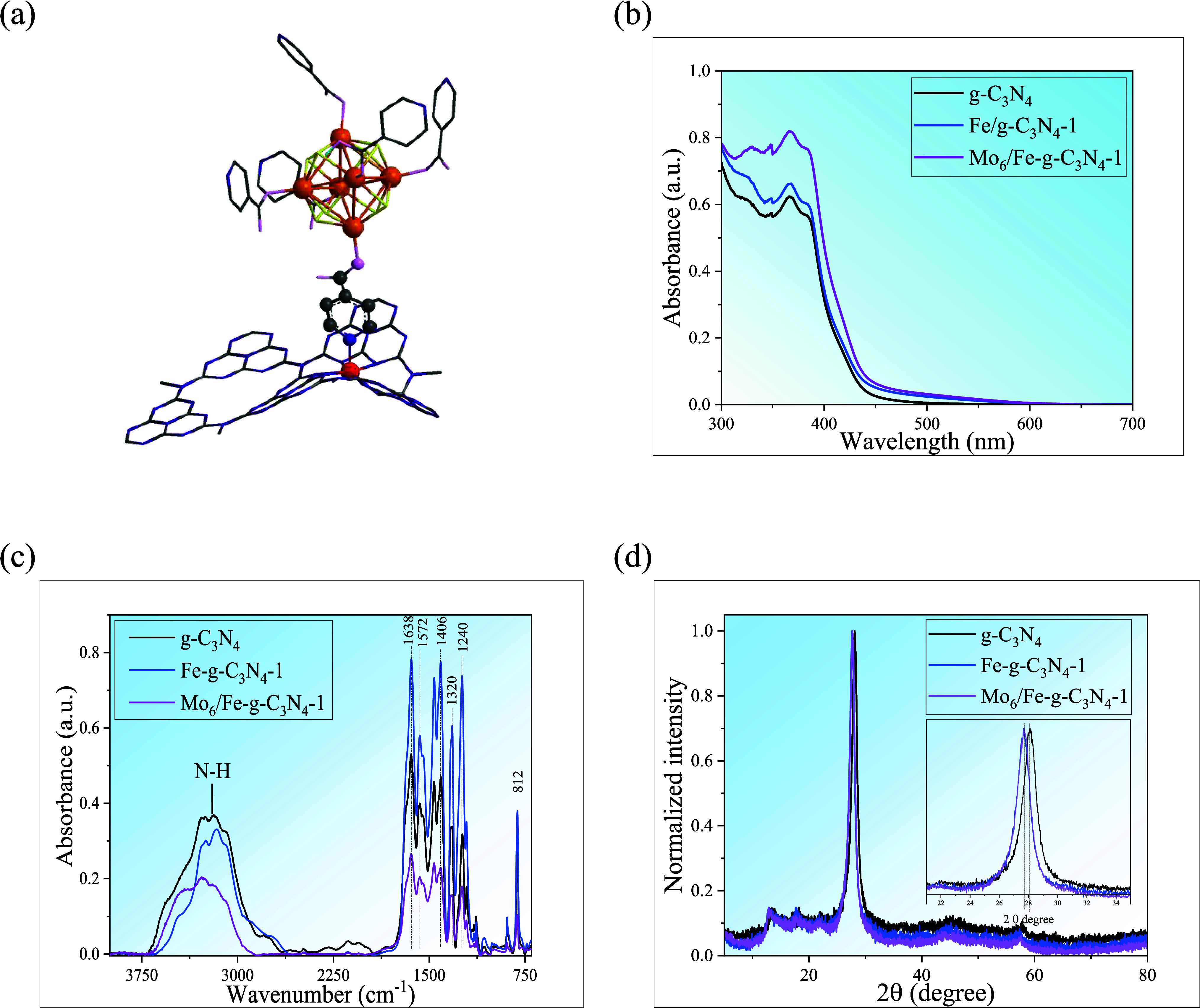
(a) Schematic representation of the interaction of Fe–N bonds between Mo_6_ and Fe-*g*-C_3_N_4_ (color codes for atoms: Fe: red, Mo, orange; I: yellow, O: malve, C: gray, N: dark blue; H atoms are omitted for clarity), (b) UV–vis DRS, (c) FTIR spectra, and (d) PXRD diffractograms of Mo_6_/Fe-*g*-C_3_N_4_-1, Fe-*g*-C_3_N_4_-1, and g-C_3_N_4_ materials.

All the nanocomposites and their precursors were characterized by additional spectroscopic, structural, analytical, and textural techniques. Considering that the graphitic materials provide similar characterization results independently of the iron content, for simplicity, the characterization of the material of greatest catalytic interest, Fe-*g*-C_3_N_4_-1 and Mo_6_/Fe-*g*-C_3_N_4_-1, is described next.

The UV–vis DRS of the Mo_6_/Fe-*g*-C_3_N_4_-1 material (Figure 1b) shows the characteristic fingerprint of the graphitic precursors, with no absorption of the Mo_6_ cluster detected (Figure S1). However, a red shift of the Mo_6_/Fe-*g*-C_3_N_4_-1 bands, and a slight increase in intensity, could be associated with the presence of the hexametallic cluster units, as was described in solution for similar systems with metal-pyridino linker–Mo_6_ coordinative interactions.^33,34^ The band gap energy (*E*_g_) was calculated by the Tauc method and, taking into consideration the direct allowed transitions of carbon nitride, the values for g-C_3_N_4_, Fe-*g*-C_3_N_4_-1, and Mo_6_/Fe-*g*-C_3_N_4_-1 were 2.67, 2.62, and 2.61 eV, respectively (Figure 1b), indicating that the incorporation of Fe and Mo_6_ allows for improvement of visible light absorption ability.^38^

The FTIR absorption spectra of the graphitic nanomaterials (Figure 1c) show a broad band between 3700 and 3000 cm^–1^, which corresponds to the vibrational mode of N–H bonds associated with amino groups and the O–H bonds of adsorbed water molecules. The peak at 1638 cm^–1^ is associated with the stretching vibrational modes of the C–N bond, while the signals at 1572, 1406, 1320, and 1240 cm^–1^ correspond to aromatic C–N stretching vibrations. The peak at 890 cm^–1^ indicates the out-of-plane bending mode of N–H, and the peak observed near 810 cm^–1^ represents the triazine ring breathing mode (Figure S2). The characteristic signals of the Mo_6_ cluster are not detected due to the low content and overlap with the graphitic IR signals (Figure S2). The similar vibrational fingerprints between the graphitic materials suggest that the g-C_3_N_4_ structure is retained after incorporation of Fe and Mo_6_. To verify the presence of clusters in Mo_6_/Fe-*g*-C_3_N_4_-1, Raman spectra of this hybrid and the Mo_6_ precursor were recorded in the characteristic Raman region of the octahedral molybdenum clusters.^27,41,42^ Figure S3 shows intense bands with Raman shifts at 155, 198, 255, 310, and 420 cm^–1^ associated with the cluster-specific bonds Mo–Mo, Mo–I, and Mo–O.^28,41,42^ Unfortunately, no signals were detected in the Mo_6_/Fe-*g*-C_3_N_4_-1 spectrum, probably due to the low concentration and/or the high luminescence of the Mo_6_ compound.

The PXRD patterns of the Mo_6_/Fe-*g*-C_3_N_4_-1 photocatalyst were measured and compared with the pristine materials. The diffractogram of g-C_3_N_4_ (Figure 1d) shows two typical signals at 12.4° (2θ), which is associated with the (100) plane and is due to the repetition of heptazine rings, and at 27.7°(2θ), which corresponds to the (002) plane and is associated with the stacking of the conjugated aromatic system, as observed in graphite.^43^ For the Fe-doped and the Mo_6_/Fe-*g*-C_3_N_4_-1 materials (Figure 1d), the peak of the (002) plane is little shifted to lower angles (0.2°) with respect to the support, which reveals a certain lattice disorder of g-C_3_N_4_ due to the presence of Fe, generating a less dense packing in the crystal lattice.^13^ This change is mainly associated with the interstitial positioning of Fe ions in carbon nitride by chemical coordination with the free electron pairs of the nitrogen atoms in its structure.^13^ Due to the low concentration of the Mo_6_ cluster deposited on the Fe-*g*-C_3_N_4_ surface, there are no diffraction peaks of crystalline Mo_6_ (Figure S4a) and no changes in the diffraction patterns of the graphitic support.^34^

The morphology and microstructure of graphitic materials were investigated by FESEM, and the results are shown in Figure 2. The pristine g-C_3_N_4_ support exhibits a petal morphology and, in detail, looks like small sheets placed on top of each other. The same morphology is observed for Fe-*g*-C_3_N_4_-1 and Mo_6_/Fe-*g*-C_3_N_4_-1, as expected, considering the small changes in the surface area among the nanomaterials. A greater stacking of the sheets is detected for Mo_6_/Fe-*g*-C_3_N_4_-1, which agrees with a slight decrease in surface area after Mo_6_ immobilization. Mo and Fe are not detected due to their low concentration, whereas EDS analyses confirm the presence of Cl coordinated to iron atoms.

**Figure 2:**
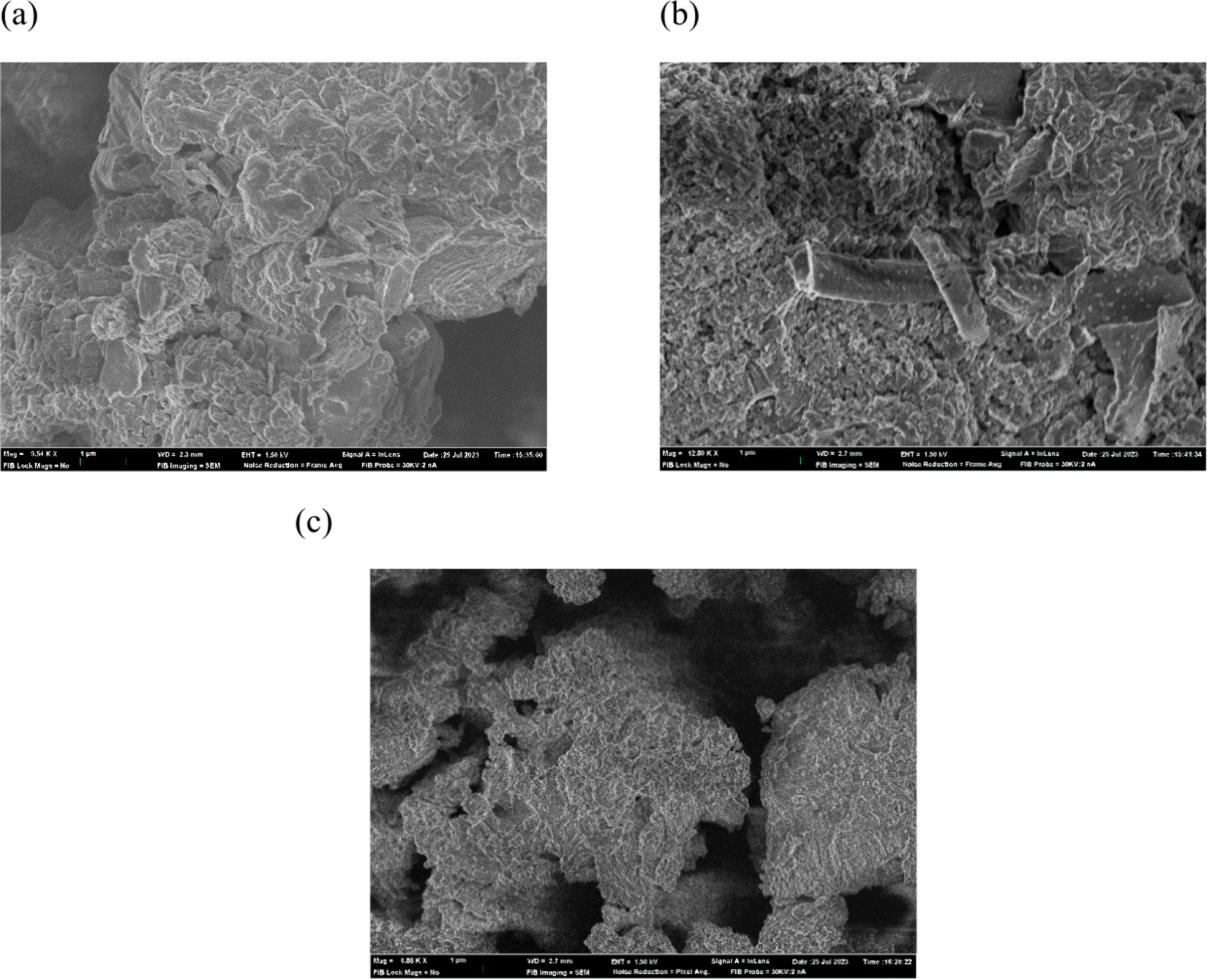
FESEM images of (a) g-C_3_N_4_ (b) Fe-*g*-C_3_N_4_-1, and (c) Mo_6_/Fe-*g*-C_3_N_4_-1.

XPS analysis of Mo_6_/Fe-*g*-C_3_N_4_-1 was performed to verify the electronic nature of the metals. Figure 3a presents the survey scan XPS spectra of the Mo_6_/Fe-*g*-C_3_N_4_-1sample, which shows the characteristic peaks of C, O, N, Fe, I, and Mo at 285, 535, 398, 710, 619, and 230 eV, respectively. The resolution of the C 1s region (Figure 3b) shows two primary peaks with band energy values of 289.7 and 286.5 eV. The first one corresponds to the sp^2^ C–O and tri-s-triazine structures N–C=N bonds of the support, and the second one can be associated with sp^2^ C–C of apical ligands in the Mo_6_ cluster and with the C absorbed on the sample surface.^44^ Figure 3c shows the Fe 2p region, and in this, two signals located at 711 and 726 eV can be observed, which correspond to the signals Fe 2p_3/2_ and Fe 2p_1/2_, respectively. These results confirm the presence of Fe in the material as Fe^3+^. The Mo 3d region (Figure 3d) displays the typical signals of Mo^2+^ at 230 and 233.5 eV, corresponding to Mo 3d_5/2_ and Mo 3d_3/2_, respectively. The I 3d region (Figure S5) shows the characteristic doublet of I^–^ at 619.5 and 631.3 eV, corresponding to I 3d_5/2_ and I 3d_3/2_, respectively. Together, these results confirm the presence of the {Mo_6_I_8_}^4+^ cluster core on the surface of Fe-*g*-C_3_N_4_.^45^

**Figure 3:**
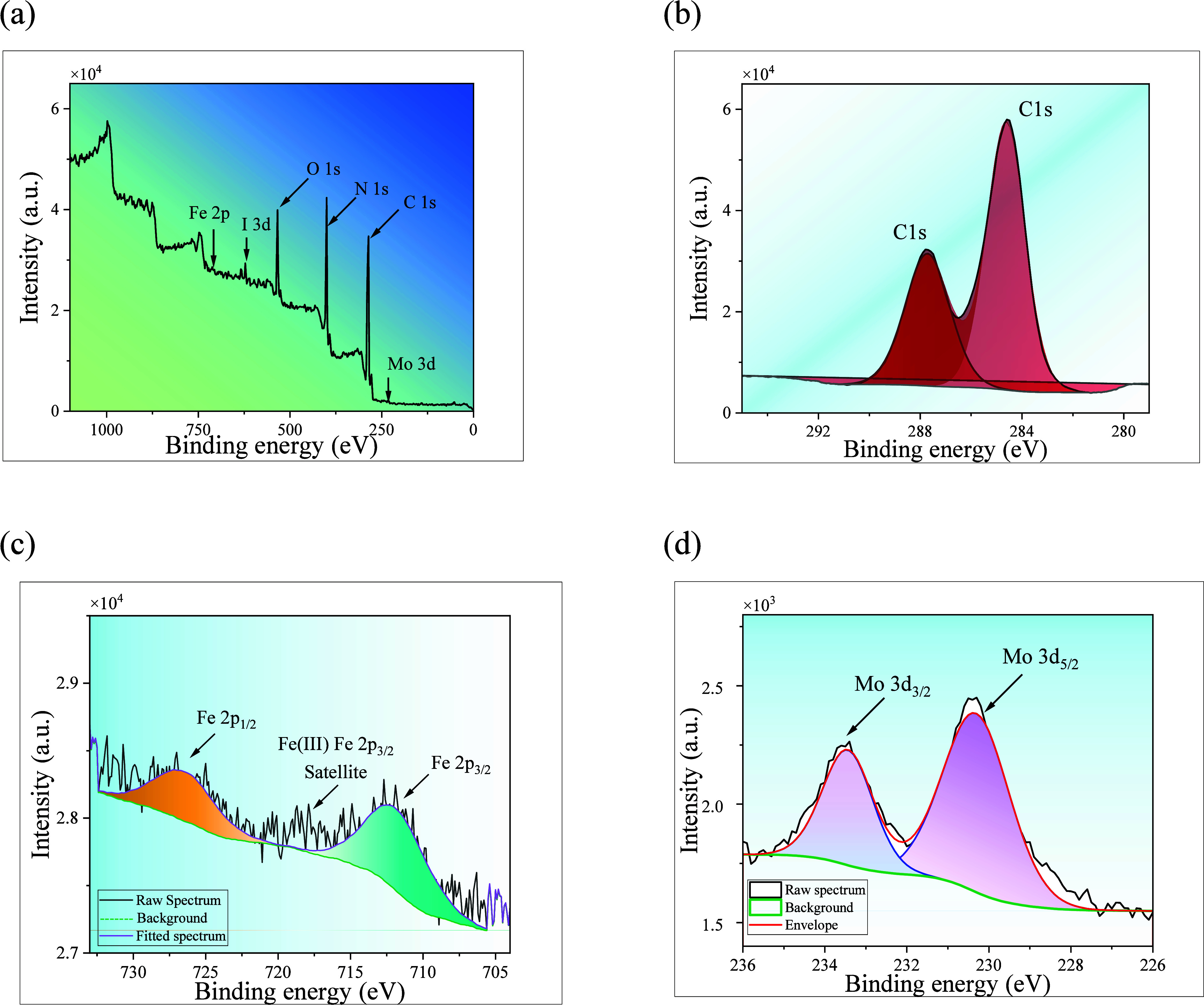
XPS analysis of the Mo_6_/Fe-*g*-C_3_N_4_-1photocatalyst: (a) full survey spectrum, (b) C 1s, (c) Fe 2p, and (d) Mo 3d.

### 2.2 Photophysical Properties

Photoluminescence studies were carried out to elucidate possible mechanisms of energy and electron transfer between the Mo_6_ cluster and the Fe-*g*-C_3_N_4_ support under illumination conditions. For this purpose, steady-state emission spectra of Mo_6_/Fe-*g*-C_3_N_4_-1 and Fe-*g*-C_3_N_4_-1 were investigated and compared with those of their precursors. Upon light excitation (λ_exc_ = 365 nm), a broad emission band appears at 480 nm in the spectra of the graphitic materials (Figure 4a). This emission is characteristic of g-C_3_N_4_ and attributed to π* transitions from the valence band (VB) to the conduction band (CB),^46^ as well as to band edge transitions associated with lone pair states of nitrogen atoms present both within and between tri-s-triazine units.^47^ The emission band of the g-C_3_N_4_ increases upon incorporation of Fe into the graphitic support, contrary to what has been described in the literature,^21,48^ suggesting a higher recombination of photogenerated charge carriers when this material is irradiated. The characteristic photoluminescence of Mo_6_ is detected in the spectra of the Mo_6_/Fe-*g*-C_3_N_4_-1 material, with an emission maximum close to that registered for the pristine cluster (705 nm, Figure S6), which is attributed to electronic transitions from triplet excited to the ground states of the hexametallic cluster unit.^49,50^ Thus, the Mo_6_/Fe-*g*-C_3_N_4_-1 material displays two characteristic emission bands of the precursors, confirming that the Mo_6_ cluster is anchored to the support.

**Figure 4:**
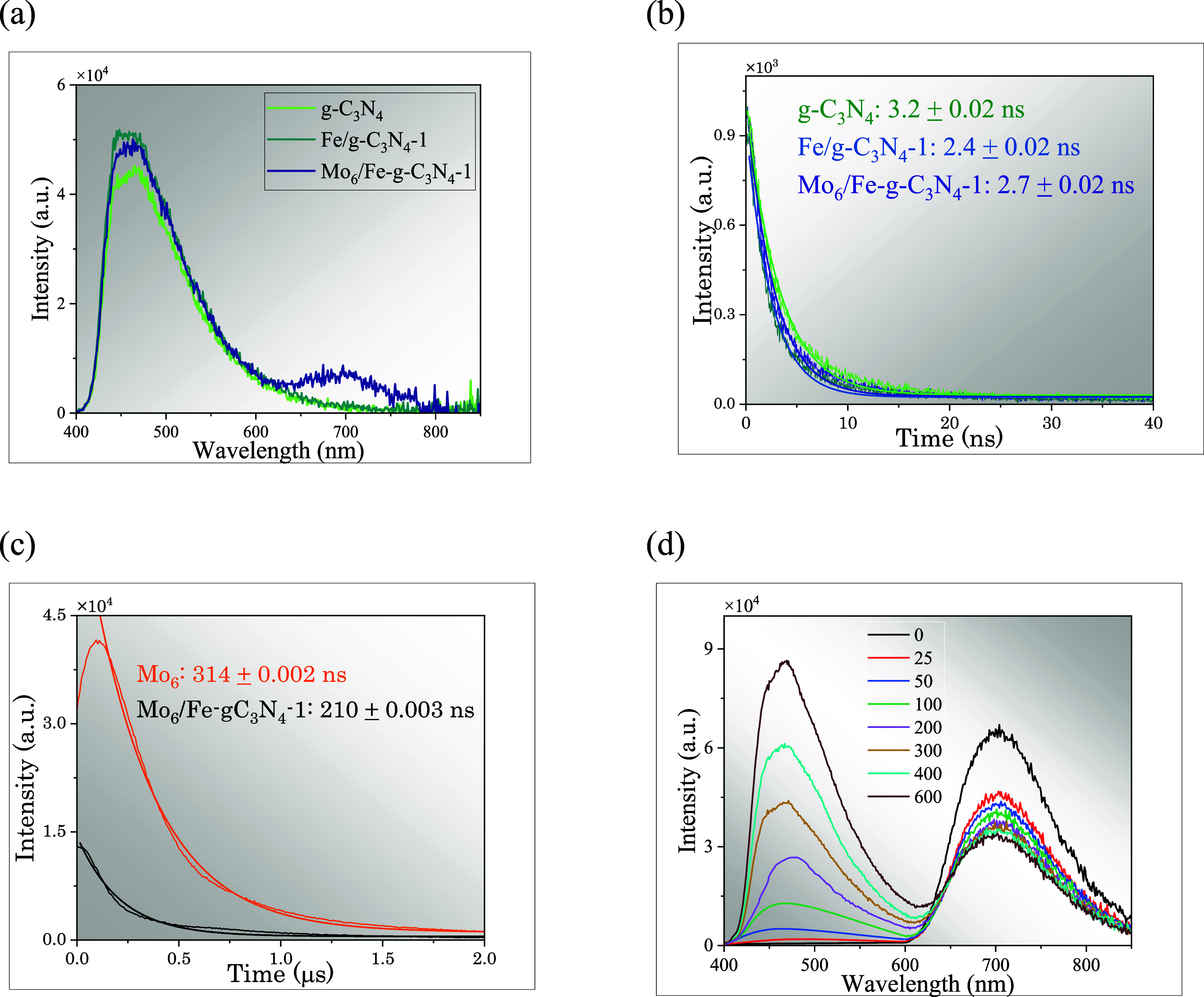
(a) Steady-state emission spectra of Mo_6_, g-C_3_N_4_, and the hybrid nanomaterials measured in acetonitrile under a N_2_ atmosphere and λ_exc_ = 365 nm; temporal decay profiles of the emission of Mo_6_, g-C_3_N_4_, and Mo_6_/Fe-*g*-C_3_N_4_-1 nanomaterials recorded at (b) λ_exc_ = 365 nm and (c) λ_em_ = 705 nm. (d) Steady-state emission spectra of Mo_6_ in acetonitrile (0.25 mg·mL^–1^) with increasing volumes (in mL) of a suspension of Fe-*g*-C_3_N_4_-1 in acetonitrile, recorded in air with excitation at λ_exc_ = 365 nm.

The emission lifetimes were determined by fitting to a first-order exponential function, as shown in Figure 4b,c. The values calculated from the temporal decay profiles of the emission of the materials, recorded at λ_exc_ = 365 nm and λ_em_ = 480 nm, reveal that the emission lifetime of g-C_3_N_4_ is 3.2 ± 0.02 ns, whereas for Fe-*g*-C_3_N_4_-1 and Mo_6_/Fe-*g*-C_3_N_4_-1, the calculated values decrease to 2.4 ± 0.02 ns and 2.7 ± 0.02 ns, respectively, indicating that the recombination of photogenerated charge carriers in the hybrid materials is slightly faster.^48^ Additionally, the emission lifetime of the Mo_6_ cluster at λ_em_ = 705 nm was determined. After the data were fitted to a first-order exponential, the calculated value was 314 ± 0.002 ns. For the Mo_6_/Fe-*g*-C_3_N_4_-1 material, the calculated lifetime decreases to 210 ± 0.002 ns. This reduction in lifetime suggests the presence of a new (or additional) nonradiative deactivation pathway for the excited state of the cluster when it is in the carbon nitride environment. This implies that charge or energy transfer from Mo_6_ to Fe-*g*-C_3_N_4_-1 may occur due to strong interactions between the two components, facilitating additional nonradiative processes. Furthermore, it becomes evident that modification of the electronic environment of the cluster through interaction with the iron-doped carbon nitride alters its emissive efficiency. To support this hypothesis, photoluminescence measurements were recorded for a solution of the Mo_6_ cluster in acetonitrile (0.25 mg·mL^–1^), to which increasing amounts of a suspension of Fe-*g*-C_3_N_4_-1 in acetonitrile were added (Figure 4d). The progressive decrease in the intensity of the Mo_6_ cluster emission band (λ_em_ = 705 nm) and the increase in the characteristic emission band of the nitride (λ_em_ = 480 nm) upon adding increasing amounts of Fe-*g*-C_3_N_4_-1 suggest that an electron/energy transfer from the Mo_6_ cluster to the Fe-doped graphitic support occurs.

Based on all of the characterization data described above, the hybrid materials are potentially useful in photocatalytic processes. This is due to the effective immobilization of the molybdenum cluster on the graphitic support, probably associated with coordinative interactions, which leads to an enhancement of the physicochemical properties of the hybrid material. As a result, the material exhibits increased radiation absorption and more efficient charge carrier separation while maintaining its textural properties unaltered.

### 2.3 Photocatalytic CO_2_RR Studies

The performance of the Mo_6_/Fe-*g*-C_3_N_4_-*n* (*n* = 1, 3, 7) nanocomposites and their precursors, g-C_3_N_4_, Fe-*g*-C_3_N_4_-*n*, and Mo_6_, in the CO_2_ reduction, was assessed using a TEOA/acetonitrile (1% v/v) mixture and gentle heating (50 °C) for 24 h of irradiation, under standard reaction conditions. In the first study, the photocatalytic activity of the precursors was assessed. The g-C_3_N_4_ material provided H_2_ and CH_4_ (256 μmol and 22 μmol·g^–1^, respectively; Figure 5a), being the H_2_ produced 2-fold the H_2_ obtained by photocatalytic decomposition of TEOA (Figure S8). No additional C-based products were detected as products of the CO_2_RR.

**Figure 5:**
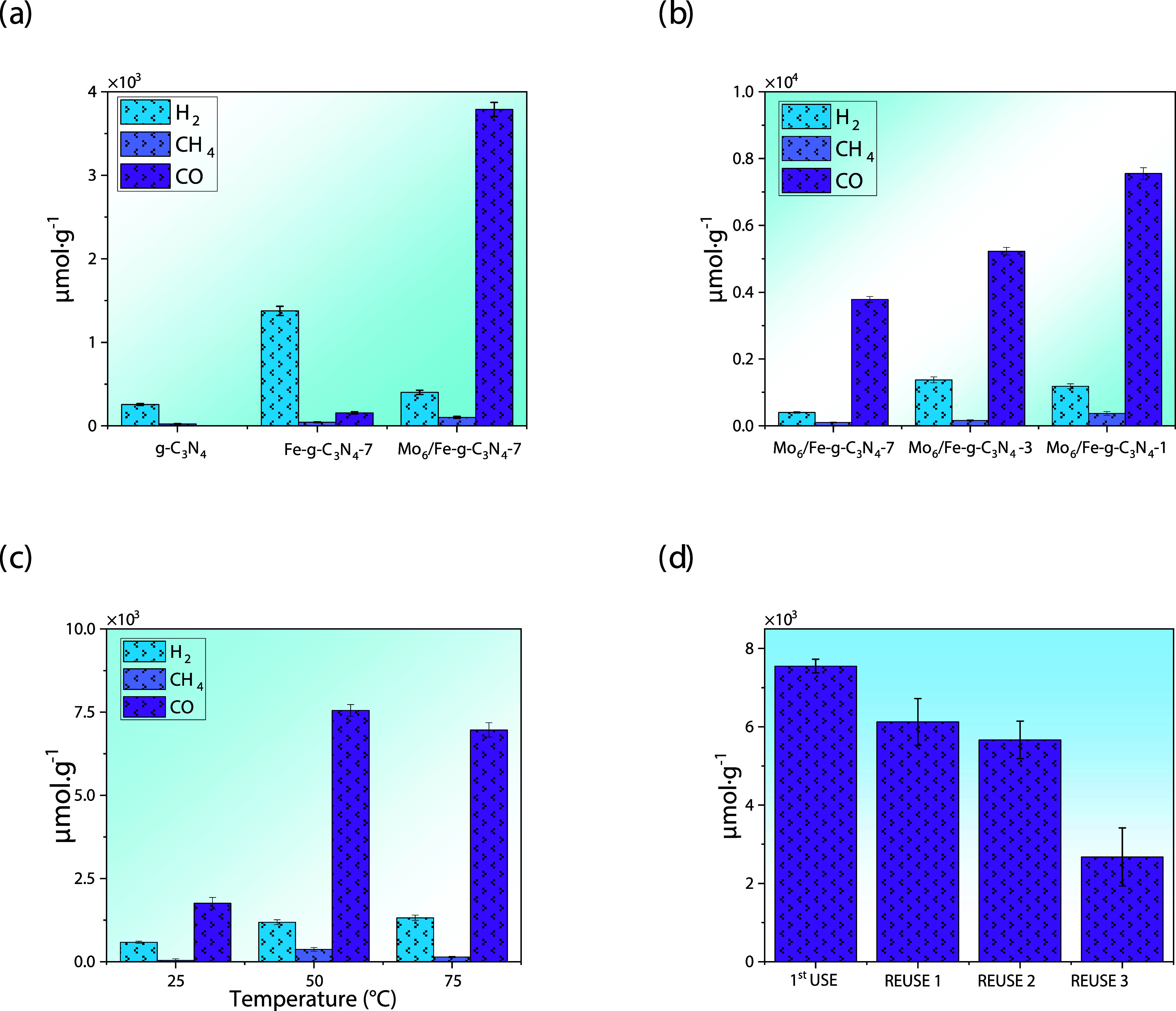
(a) Representation of products from photocatalytic CO_2_RR using g-C_3_N_4_-based photocatalysts; (b) influence of Fe content in the CO_2_RR using Mo_6_/Fe-*g*-C_3_N_4_-*n* (*n* = 1, 3, and 7) photocatalysts; (c) influence of temperature in the photocatalytic CO_2_RR promoted by Mo_6_/Fe-*g*-C_3_N_4_-1; and (d) reutilization tests of the Mo_6_/Fe-*g*-C_3_N_4_-1 photocatalyst.

Several studies have reported that the modification of g-C_3_N_4_ by incorporating an iron atom into the triazine rings enhances the photocatalytic performance of the support.^36^ Our results show that the Fe-*g*-C_3_N_4_-7 material produces H_2_, CH_4_, and CO in different amounts (1376, 43, and 184 μmol·g^–1^, respectively; Figure 5a), evidencing that the selectivity of the CO_2_RR toward CO changes with respect to g-C_3_N_4_. In addition, the efficiency in CO production is significantly higher than that reported for the same material exposed to less irradiation time (10 h) and dispersed in pure water.^21^ Studies conducted by DFT calculations showed that the VB of carbon nitride is a combination of the HOMO levels of the triazine monomer derived from Npz orbitals, while the CB consists mostly of LUMO levels of the Cpz orbitals.^37,43,51^ In consequence, the transfer of the light-generated electrons in the triazine rings to the surface for their involvement in oxidation–reduction reactions proves challenging, leading to very rapid recombination processes of the photogenerated electron–hole pairs. The positive metallic species (Fe^3+^, as evidenced by XPS) in the interstitial positions of these rings act as attractive centers for photogenerated negative charge species (e^–^), improving charge separation and increasing their average lifetime,^52^ as confirmed by photophysical studies.^21^ This results in an enhancement of the photocatalytic efficiency of the materials in the presence of Fe. Thus, the trapping of electrons by Fe^3+^ species in Fe-*g*-C_3_N_4_ increases the quantity of available electron holes for oxidation processes, leading to a higher production of H_2_ as a product of TEOA oxidation.

Once Mo_6_ is supported onto Fe-*g*-C_3_N_4_-7, the resulting Mo_6_/Fe-*g*-C_3_N_4_-7 material achieves a maximum CO production of 3600 μmol·g^–1^ (Figure 5a), making it a highly efficient reaction (150 μmol·g^–1^·h^–1^). The high selectivity toward this product (87.8%) changed drastically with respect to the selectivity of the support. To give more insight into the role of the cluster compound, the photocatalytic activity of the molecular Mo_6_ cluster toward CO_2_ was tested under the same experimental conditions but in a homogeneous phase. After 24 h, the formation of CO, CH_4_, and H_2_ was observed, with CO being again the preferential product formed (Table S2). In contrast to the photophysical and photocatalytic characteristics of Mo_6_/Fe-*g*-C_3_N_4_, the isolated Mo_6_ clusters act as a photosensitizer and as a photocatalyst, confirming the dual role of the hexametallic cluster.^25,53^

To enhance the performance of Mo_6_/Fe-*g*-C_3_N_4_-7 in the CO_2_RR, lower concentrations of Fe were tested on Mo_6_/Fe-*g*-C_3_N_4_-*n* (*n* = 1, 3) while keeping the reaction conditions unchanged. As shown in Figure 5b, the value of CO obtained (7752 μmol·g^–1^) with the Mo_6_/Fe-*g*-C_3_N_4_-1 catalyst shows a significant improvement with respect to the other two Mo_6_/Fe-*g*-C_3_N_4_ nanocatalysts tested and to most of the graphitic hybrid systems reported in the literature (Table S2), demonstrating that this three-component system is a benchmark in CO_2_-to-CO conversion among carbon nitride composites reported until date. The decrease in the amount of iron in the photocatalyst progressively enhances CO production with a slight decrease in selectivity (from 88 to 80%). This result supports the idea that by reducing the number of Fe^3+^ electron-trapping centers, more photogenerated electrons become available to promote reduction processes. However, the presence of Fe^3+^ in this material is crucial, as demonstrated by UV–vis DRS spectroscopy (Figure 1b) since the absorption spectrum of the material shifts slightly toward the visible region and, similarly, the energy band gap in the modified materials decreases compared to g-C_3_N_4_. Additionally, Fe^3+^ atoms act as anchoring points for the Mo_6_ cluster, which functions as a photosensitizer, allowing a greater number of photons to be captured by the system. Furthermore, considering the concentration of the Mo_6_ cluster in the material, it is assumed that the vast majority of Fe^3+^ atoms serve as anchoring points for the cluster. The catalytic results obtained can also be supported due to the large specific surface area calculated for the modified materials, which can promote the adsorption, desorption, and diffusion of products and reactants, favoring the photocatalytic performance.^35^

Once demonstrated that Mo_6_/Fe-*g*-C_3_N_4_-1 shows the best efficiency for CO production, this nanomaterial was selected to study the effect of temperature (25, 50, and 75 °C) on the catalytic performance. The results obtained (Figure 5c) at room temperature show that the catalyst reduces its effectiveness in CO production to one-fourth of the efficiency achieved at 50 °C. However, when the temperature is increased to 75 °C, the efficiency and selectivity of the material remain unchanged. The increase in the photocatalytic activity of the materials with the temperature rise is mainly because of supplying more energy to the system, the molecules in the medium move faster, resulting in a higher collision frequency, and these collisions occur with greater force. This would mean that the reactants are more likely to overcome the activation energy barrier and transition into product formation. However, when the temperature in this system is increased to 75 °C, the CO production does not improve significantly. This is because the CO_2_ conversion is favored at lower temperatures due to the thermodynamic limit.^54^ The produced CO originating from CO_2_ was verified by GC-MS through the execution of an experiment with C-13 labeled carbon dioxide under the same reaction conditions at 50 °C (Figures S9 and S10). In the results, characteristic signals of 13-CO at 13, 16, and 29 *m*/*z* are observed. Additionally, it can be noted that the formed methane also originates from 13-CO_2_, as indicated by peaks at 14, 15, 16, and 17 *m*/*z*, corresponding to the molecular fractionation signals for mono-, bi-, tri-, and tetrasubstituted alkanes, respectively.

Reuse experiments were done (Figure 5d) and showed that Mo_6_/Fe-*g*-C_3_N_4_-1 loses activity after the first use, reducing the amount of CO produced by 19% for the first reuse and 25% for the second. This behavior may be due to aging of the material with prolonged exposure to light or surface contamination derived from the oxidation byproducts of TEOA present in the reaction system. However, it is important to highlight that leaching tests conducted by ICP measurements showed the absence of Fe or Mo in the solution, indicating the physical stability of the material after the reaction. Additionally, photoluminescence measurements carried out on the Mo_6_/Fe-g-C_3_N_4_-1 material after three cycles of use (Figure S11) showed that the emission band of the cluster decreased in intensity, suggesting partial decomposition of the Mo_6_ cluster, in agreement with the decrease in the material’s catalytic efficiency along the reuses.

### 2.4 Proposed Photocatalytic Reaction Mechanism

The selectivity of the CO_2_RR reaction could be due to the preferential CO_2_ to CO transformation, from a thermodynamic standpoint, which involves 2 electrons instead of 8 electrons required for CH_4_ production, as shown in eqs 1 and 2:^10,52^

$${\mathrm{CO}}_{2}+2H^{+}+2e^{-}\to \mathrm{CO}+H_{2}O$$ 1

$${\mathrm{CO}}_{2}+8H^{+}+8e^{-}\to {\mathrm{CH}}_{4}+2H_{2}O$$ 2

From the perspective of the reaction mechanism, the high efficiency of Fe-*g*-C_3_N_4_-7 toward CO production is attributed to the coordinative interaction between the pyridyl group of the apical ligand of Mo_6_ and the iron atom, as illustrated in Figure 5b. The interaction of N–Fe bonds between the apical ligands of the Mo_6_ cluster and the Fe^3+^ atom incorporated into the g-C_3_N_4_ was assumed based on studies of similar systems that have demonstrated this behavior.^33,34^ Two hypotheses are proposed to explain the high efficiency and selectivity of the hybrid in the reaction: (i) considering the molybdenum cluster as a photosensitizer; its anchoring would allow the transfer of photogenerated electrons to the Fe^3+^ atoms integrated into the graphitic structure, providing a greater number of electrons available to participate in reduction processes; (ii) considering the cluster as both a photosensitizer and a catalytic center, whereby the photogenerated electrons concentrate on the cluster unit, similar to what occurs in the molecular Mo_6_ cluster, while holes are transferred to the support. In the first hypothesis, the Fe^3+^ atoms are regarded as the catalytic centers, whereas in the second, the cluster unit itself is considered the active center, given that the selectivity of the transformation relative to the molecular Mo_6_ cluster is maintained.

Keeping these hypotheses in mind, and considering the mechanism proposed by Zhang and co-workers for Fe-*g*-C_3_N_4_ systems in the CO_2_ photocatalytic transformation,^21^ we present the possible mechanism reaction involved in this research (Figure 6). Once the illumination of the material induces the electron excitation from the HOMO to the LUMO in the Mo_6_ cluster, the photogenerated electrons migrate to the CB of the iron-doped carbon nitride, improving the separation of the charge carriers (as a sensitizer mechanism).^6^ At the same time, the photogenerated holes in the hybrid material react with TEOA in solution to produce TEOA^+^, which could react with the CO_2_ adsorbed to form COOH^·^ and TEOA, in a similar manner to what was proposed by Zhang and co-workers (in their case, the molecule oxidized is water to OH^·^).^21^ Then, the next step involves the cleavage of the C–O bond in COOH^·^, generating CO and another OH^·^. The OH^·^ species are involved in the TEOA photooxidation, ultimately leading to the production of CO_2_ and H_2_ as main products. Besides, it is important to mention that all the photochemical process described before occurs at the active site of Fe, and this is due to the fact that the spin density is distributed mainly on the Fe atom in the interstitial positions of g-C_3_N_4_.^21^ On the basis of this scheme (Figure 6), the Mo_6_ cluster would act as a photosensitizer and promote the electron injection into the iron reactive sites of the graphenic support.

**Figure 6:**
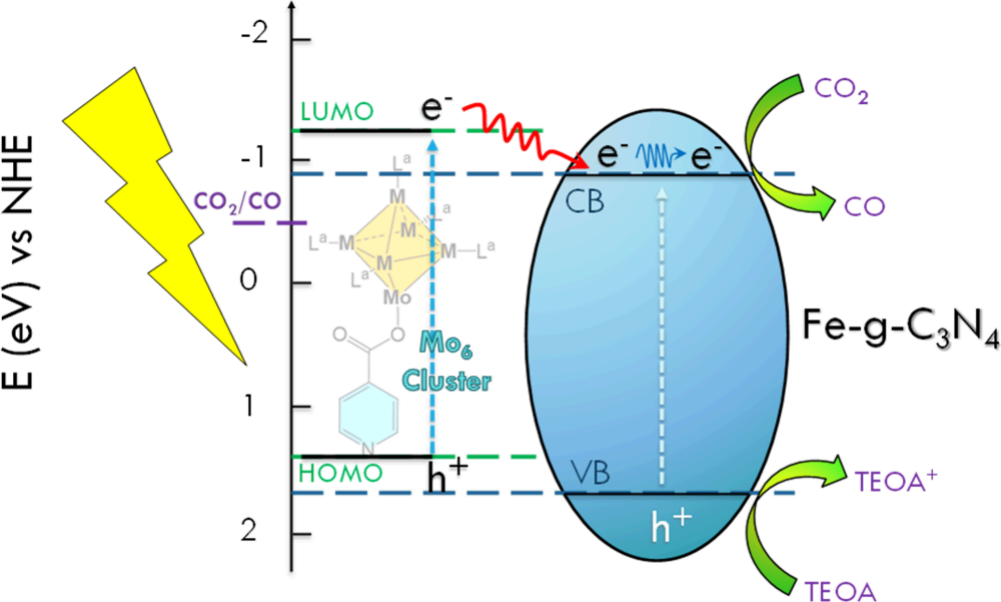
Schematic representation of the reaction mechanism for photoreduction of CO_2_ to CO using Mo_6_/Fe-*g*-C_3_N_4_ hybrid materials and TEOA as a sacrificial agent.

## 3 Conclusions

The immobilization of octahedral molybdenum clusters, bearing iodido and carboxylato ligands with terminal pyridyl groups, onto iron-doped carbon nitrides was effectively carried out using an impregnation method. The textural, structural, morphological, and spectroscopic characterization of the resulting nanostructured materials (Mo_6_/Fe-*g*-C_3_N_4_) shows good consistency with the physicochemical properties previously described for both the octahedral clusters and carbon nitride materials, individually.

The studies confirmed that the exfoliation processes applied to the carbon nitride led to an improvement in the specific surface area of the pristine carbon nitride. The incorporation of Fe^3+^ and the molybdenum cluster into the g-C_3_N_4_ structure causes a negligible decrease in the material’s surface area, which is not considered critical for catalytic applications and is attributed to pore blocking in the graphitic material. It was verified that the estimated Fe mass ratio is similar to that used during the doping reaction, while the maximum Mo_6_ content reached 0.8% w/w. The incorporation of iron and molybdenum into the g-C_3_N_4_ support leads to enhanced light absorption properties and a slight reduction in band gap energy (from 2.67 to 2.61 eV). The electronic properties of the molybdenum and iron precursors are preserved after their integration into the carbonaceous material. The photophysical properties of the Mo_6_/Fe-*g*-C_3_N_4_ hybrid materials show that both components retain their emission characteristics in the composite material, confirming the presence of the cluster anchored to the support. However, the decrease in the emission lifetime of the Mo_6_ cluster upon integration into the carbon nitride suggests an electronic interaction between the two components, which promotes nonradiative deactivation processes possibly attributable to charge or energy transfer toward the support.

The photocatalytic evaluation of the Mo_6_/Fe-*g*-C_3_N_4_ nanomaterials in the CO_2_ valorization demonstrated high efficiency and selectivity for CO in acetonitrile in the presence of TEOA as a sacrificial agent. While the incorporation of Fe (7% w/w) into the g-C_3_N_4_ structure leads to the formation of H_2_, CO, and CH_4_, the addition of the Mo_6_ cluster to the Fe-*g*-C_3_N_4_-7 material improves CO production, yielding 3600 μmol·g^–1^ catalyst after 24 h of reaction, thus indicating that the CO production efficiency of the tricomponent material increases by a factor of 20 once the Mo_6_ cluster is grafted on the iron-doped graphenic surface. Furthermore, the presence of the metal cluster enhances the selectivity of this transformation to 88%. Interestingly, CO production further increases to approximately 2-fold (7752 μmol·g^–1^) when the Fe content is reduced to 1% under the same reaction conditions, thereby verifying that the Mo_6_/Fe-*g*-C_3_N_4_-1 nanostructured material is the most active and efficient catalyst, with lower metal content. This phenomenon is attributed to the more homogeneous distribution of Fe in the material and the greater availability of photogenerated electrons to participate in the reduction processes. Neither decreasing nor increasing the temperature (to 25 or 75 °C) compared to the initial reaction conditions (50 °C) led to improved CO production. Produced CO originates exclusively from the photoreduction of CO_2_, as confirmed by ^13^C labeling experiments. The proposed catalytic reaction mechanism implies electron transfer from the Mo_6_ cluster to the Fe reactive sites of the graphenic support.

## Supplementary Material



## Notes

Experimental details (synthesis, characterization, and catalytic processes); materials (specific surface area, ICP, UV–vis, FTIR, Raman, PXRD, XPS, NMR, photoluminescence); CO_2_RR products (GC and GC-MS); and comparison of the catalytic performance with the literature (PDF)
