# Supplementary material for: Efficient and Selective Photocatalytic Transformation of CO2 to CO with Mo6 Clusters Supported on Fe-Doped g‑C3N4
Source: ACS Appl Energy Mater. 2025 Oct 8;8(20):15146–56. doi: 10.1021/acsaem.5c02019 (PMC12570109; doi:10.1021/acsaem.5c02019)
Supplement: Supplementary file 1 [file ae5c02019_si_001.pdf]

Supporting Information

Efficient and Selective Photocatalytic Transformation of CO_2 to CO with Mo_6 Clusters Supported on Fe-Doped g-C_3N_4

*Jhon S. Hernández and Marta Feliz**

Instituto de Tecnología Química (Universitat Politècnica de València – Agencia Estatal Consejo Superior de Investigaciones Científicas), Avd. de los Naranjos s/n, 46022 Valencia, Spain.

*Corresponding author. E-mail: mfeliz@itq.upv.es

TABLE OF CONTENTS:

1. EXPERIMENTAL METHODS...S2

2. FIGURES AND TABLES..S7

3. REFERENCES...S16

1. EXPERIMENTAL METHODS

Chemicals and materials: Melamine (99%) was provided by Acros Organics. Iron (III) chloride hexahydrate (ACS Reagent, 97%), triethanolamine (TEOA, ACS Reagent $\geq$ 99%), methanol (ACS Reagent, $\geq$ 99.8%), acetonitrile (CH_3CN, ACS Reagent $\geq$ 99 %), dichloromethane (CH_2Cl_2, ACS Reagent > 99.8%), acetone (ACS Reagent $\geq$ 99%), and diethyl ether (ACS Reagent 98%) were obtained for commercial resources (Sigma-Aldrich). Tetrahydrofuran (THF, CHROMASOLV™ Plus, inhibitor-free, for HPLC, ≥99.9%, Honeywell Riedel-de Haën) was dried and deoxygenated by passing the solvents through CuO and alumina commercial columns under nitrogen atmosphere. The ultrapure water was obtained by the Milli-Q eq 7000 Type 1 water purification system. Schlenk techniques were used for manipulations under an inert atmosphere.

Preparation of Mo_6 cluster precursors: The Mo_6 cluster precursors (namely, $A_2[Mo_6I_{14}]$ (A^+ = Cs^+ and Bu_4N^+) were prepared following reported procedures. The synthesis of $Cs_2[Mo_6I_{14}]$ was carried out following the procedure described by Meyer et al. [1] In a typical procedure, CsI (395.6 mg, 1.52 mmol), Mo (441.0 mg, 4.60 mmol), and I_2 (1168.0 mg, 4.60 mmol) were placed in a mortar in a stoichiometric ratio of 2:6:12 and ground in a glovebox under a N_2 atmosphere (total mass 2004.6 mg). The mixture was transferred to a quartz reactor and sealed under vacuum while cooled with liquid N_2. The ampoule was placed in a horizontal furnace, heated to 700 °C at a rate of 5 °C·min^{-1}, and kept at this temperature for 30 minutes. It was then allowed to cool to room temperature and gently shaken to homogenize the product. The heating and homogenization procedures were repeated three times. Subsequently, the ampoule was opened to air, the product was ground in a mortar, and 1948 mg of a red product were obtained (yield: 97%), identified as $Cs_2[Mo_6I_{14}]$. Finally, the material was stored in a desiccator. Afterwards, the synthesis of the $(Bu_4N)_2[Mo_6I_{14}]$ compound was carried out following the methodology

described by Kiracki et al. [2] $Cs_2Mo_6I_{14}$ (1000.0 mg, 0.382 mmol) and $(Bu_4N)I$ (500.0 mg, 1.35 mmol) were added to a round-bottom flask containing a dichloromethane/water mixture (1:1; 40 mL). The system was stirred constantly at 30 °C for 24 hours. Subsequently, the mixture was separated in a separatory funnel, and the organic phase (colored solution) was recovered. The solvent was evaporated using a conventional rotary evaporator, affording 980.6 mg of a reddish crystalline solid corresponding to the cluster $(Bu_4N)_2[Mo_6I_{14}]$ (yield: 90%)

Preparation of Mo_6 cluster: The synthesis of the Mo_6 compound was carried following the next procedures: NaOH (136 mg, 3.4 mmol) and isonicotinic acid (123.11 mg, 1 mmol) were added to an aqueous solution of $AgNO_3$ (169.88 mg, 1 mmol) and stirred for 30 minutes at room temperature. The resulting white residue (silver isonicotinate) was filtered off, washed with water, methanol, and diethyl ether, and dried under vacuum. The yield was 85–90%. Then, $(Bu_4N)_2[Mo_6I_{14}]$ (100 mg, 0.035 mmol) was dissolved in acetone, and a 6-fold molar equivalent of the prepared solid silver salt (57 mg, 0.246 mmol) was added. The mixture was stirred for 1 day at 40 °C in the dark. Afterwards, the resulting suspension was filtered through cellulose filter paper, and the filtrate was rotary evaporated until an orange oil was obtained. The oil was redissolved in approximately 1 mL of CH_2Cl_2, and after dropwise addition of cold diethyl ether, a precipitate formed. Finally, the product was dried under vacuum, and the procedure was repeated to obtain a crystalline solid, identified as Mo_6. ^1H NMR (MeOD, 25 °C, δ ppm) for $Mo_6I_8O_{12}N_8C_{68}H_{96}$: 0.93 (t, normalized to 24 protons of CH_3 groups of Bu_4N^+), 8.57–8.53 (m, ortho-protons of isonicotinato ligands), 7.81 7.76 (m, meta-protons of isonicotinato ligands) (Fig. S7). The yield was 76 mg (0.027 mmol, 77%).

Preparation of g-C_3N_4: The synthesis and exfoliation of g-C_3N_4 was done following similar procedures reported by Todorova et al.[3] Thermal polycondensation of melamine adding 2 g in a porcelain crucible (50 mL) was achieved by heating up to 550 ºC for 3 h in air, with a heating rate of 5 ºC·min^{-1}, followed by natural cooling. The resulting material was exfoliated 3 times

by consecutive thermal treatment (550 ºC, 3 h). The entire mass of the product obtained in each of the stages of exfoliation was used (g-C_3N_4: 0.86 g; 1º exfoliation: 0.49 g; 2º exfoliation: 0.26 g; 3º exfoliation: 0.120 g).

Preparation of Fe-g-C_3N_4 materials: For the preparation of Fe-g-C_3N_4, we follow the method reported by Le Thi Mai et al.[4] In a 250 mL round bottom flask, 200 mg of g-C_3N_4 was dispersed in 50 mL of Milli-Q water magnetically stirring for 30 min and then ultrasonicating for 1 h to obtain a suspension of the precursor material. A suitable amount of iron (III) chloride hexahydrate corresponding to 1.5, 3.0 and 7.0 % (w/w) of Fe (atomic) was added to the above solution and stirred overnight at 90 ºC. Finally, the solution was cooled naturally, filtered by a nylon filter (pore size: 0.44 mm), washed, adding first water Milli-Q, and second, with methanol, and dried at 80 ºC for at least 6 h. The material was stored in a desiccator and denoted as Fe-g-C_3N_4-1, Fe-g-C_3N_4-3 and Fe-gC_3N_4-7, corresponding to the Fe content.

Preparation of Mo_6/Fe-g-C_3N_4 nanohybrids: In a round bottom flask, a suspension of the Fe-g-C_3N_4 material in THF (150 mg in 100 mL) was prepared was sonicated for 1h. To the resulting mixture the Mo_6 (50 mg) compound was added under argon atmosphere and subsequently, the suspension was heated at 40 ºC overnight with continuous stirring. The solid was recovered by filtration, washed with methanol and dried at 80 ºC for at least 6 h.

Instrumentation: The determination of atomic metals if the materials was performed through an inductively coupled plasma (ICP) atomic spectrometer, after aqua regia digestion of the solid materials at 180 °C, 24 h in reflux. The samples were measured in a Varian 715 spectrometer (Palo Alto, CA, USA). The specific surfaces of the materials were measured using the ASAP 2020 porosimetry instrument, and considering the Brunauer-Emmett-Teller (BET) methods. The solids were previously dried at 120 ºC and after degassed at 250 ºC for ca. 6 h. The UV-vis-NIR diffuse reflectance spectra (DRS) were recorded in the 200 to 2000 nm range using a

Varian Cary 5000 spectrophotometer. The powder X-ray diffraction (PXRD) data were obtained from solid materials with a PANaytical Cubix-Pro diffractometer equipped with a PANalytical X'Celerator detector. This instrument employed monochromatic CuKα X-ray radiation (L1 = 1.5406 Å, L2 = 1.5444 Å, I2/I = 0.5) and a tube voltage and intensity of 45 kV and 40 mA, respectively. It uses a variable slit with an irradiated sample area of 5 mm and the goniometer arm length is 200 mm. The diffractogram of the powder samples was obtained at room temperature in 2θ range of 5 – 90°. Fourier transform infrared spectroscopy (FTIR) spectra were registered on KBr pellets with a Nicolet 8700 Thermo spectrometer (ThermoFisher Scientific, Waltham, MA, USA). Raman spectra of solid materials were measured with a Renishaw "Reflex" spectrometer (Wotton-under-Edge, U.K.), equipped with an Olympus optical microscope. The composition and morphology of materials were measured by field emission scanning electron microscopy (FESEM) using a ZEISS model ULTRA55 FESEM coupled to an Oxford Instruments energy dispersive X-ray (EDS) detector. X-ray photoelectron spectroscopy (XPS) spectra of materials were measured using a SPECS spectrometer with a Phoibos 150 MCD-9 detector and a monochromatic Al Kα X-ray source (1486.6 eV). The spectra were obtained with an analyzer pass energy of 50 eV, an X-ray power of 100 W, and an operating pressure of 10^{-9} mbar. Spectrum analysis was performed using the CASA software. In all cases, the binding energies were calibrated with respect to the C 1s peak at 284.5 eV. The photoluminescence measurements were recorded in acetonitrile using an FLS1100 spectrofluorometer from Edinburgh Instruments, equipped with a 450 W xenon lamp and a double monochromator for excitation and emission, coupled to an InGaAs detector. Lifetime measurements were carried out using the same equipment, fitted with a pulsed LED as the excitation source (405 nm) and a time-correlated single photon counting (TCSPC) system as the detector. Solution ^{1}H nuclear magnetic resonance (NMR) experiments were recorded on a Bruker AVANCE 300 MHz spectrometer.

Molecular hydrogen and methane production were monitored by gas chromatography (GC) on the Agilent 490 Micro GC System. This instrument was equipped with a conductivity detector (TCD) and two channels, the first equipped with a column coated with a zeolite molecular sieve (CP-Molsieve 5 Å, Agilent J&W) to identify H_2, O_2 and N_2, and the other with a Pore Plot Q Column to analyze CO_2, CH_4 and short-chain hydrocarbons. Argon was selected as carrier gas (flow rate: 5 mL·min^{-1}). The inlet and detector temperatures in the GC run were 110 °C and 220 °C, respectively, and the isothermal oven temperature profile was set at 62 °C with an initial column pressure of 15 psi. For the quantification of CO, an Agilent 7890A GC system, equipped with a Carboxen®−1010 PLOT Capillary GC Column and a TCD detector, was used. Helium was selected as carrier gas (flow rate: 3.3 mL·min^{-1}). The inlet and detector temperatures were 228 °C and 230 °C, respectively. The heating ramp started at 35 °C, held for 8 min, and then increased to 225 °C (30 °C·min^{-1}, holding for 20 min). For the experiments with $^{13}CO_2$, an Agilent 6890N gas chromatograph equipped with an HP5-M5 capillary column (30 m × 250 μm) and coupled to an Agilent 5973N mass spectrometer was used. Helium was used as the carrier gas at a constant flow rate of 1.2 mL·min^{-1}. The column temperature for the analyses started at 50 °C for 2 minutes, then increased to 280 °C at a heating rate of 30 °C·min^{-1} and was held for 15 minutes.

Photocatalytic CO_2RR experiments: The photocatalytic reactions were carried out in acetonitrile and TEOA (1 % v/v). The content of TEOA sacrificial was optimized to avoid its decomposition under reaction conditions (see Supporting information). The photoreactor was a cylindrical quartz reactor (volume: 55 mL) covered with a heating blanket and thermocouple to keep temperature controlled. The photocatalysts tested were suspended in the solution, (5 mg in 20 mL) sonicated during 5 min and loaded into the reactor vessel; in the following, the reactor was purged with argon (5 min) and then with CO_2 (5 min) at 0.5 bar as initial pressure. The vessel was irradiated for 24 h with a Hamamatzu Xe lamp with a spotlight placed at 1 cm above

the reactor surface and under stirring. The gas phase samples (1000 μL) were collected with a Hamilton syringe and injected into the Micro GC spectrometer and 100 μL into the GC-TCD system. The molecular gases were calculated taking in account the peak area and his corresponding concentration using the standard calibration curve as reference. The micromoles of H_2, CO and CH_4 produced were calculated using the ideal gas law (n = PV/RT).

Control tests using CO_2 light and the reaction TEOA/acetonitrile (1, 5 and 20 % v/v) solutions were done in the standard conditions. Control experiments done for the pristine and hybrid materials (Table S1) confirmed the lowest or inexistent amounts of gas production obtained. Reuse experiments were carried out for three cycles under the same conditions as the above experiments. The photocatalysts were recovered by filtration and all the material was resuspended in a new solution with the same initial conditions. This procedure was repeated for each reuse cycle. The percentage of the gas amount produced was calculated with respect to the values obtained in the first use.

2. FIGURES AND TABLES

Table S1. Specific surface and atomic iron and molybdenum content determined in the graphitic materials.

**Material**	**Specific surface area ($m^2 \cdot g^{-1}$)**	**Metal content (% w/w)**	
**g-C_3N_4 initial**	11.52	-	
**g-C_3N_4 exfoliated**	118.14	-	
**Fe-g-C_3N_4-1**	112.7	Fe: 1.0	
**Fe-g-C_3N_4-3**	114.9	Fe: 2.6	
**Fe-g-C_3N_4-7**	112.6	Fe: 7.5	
**Mo_6/Fe-g-C_3N_4-1**	101.8	Fe: 0.9	Mo: 0.20
**Mo_6/Fe-g-C_3N_4-3**	109.73	Fe: 2.3	Mo: 0.16
**Mo_6/Fe-g-C_3N_4-7**	100.19	Fe: 7.4	Mo: 0.18

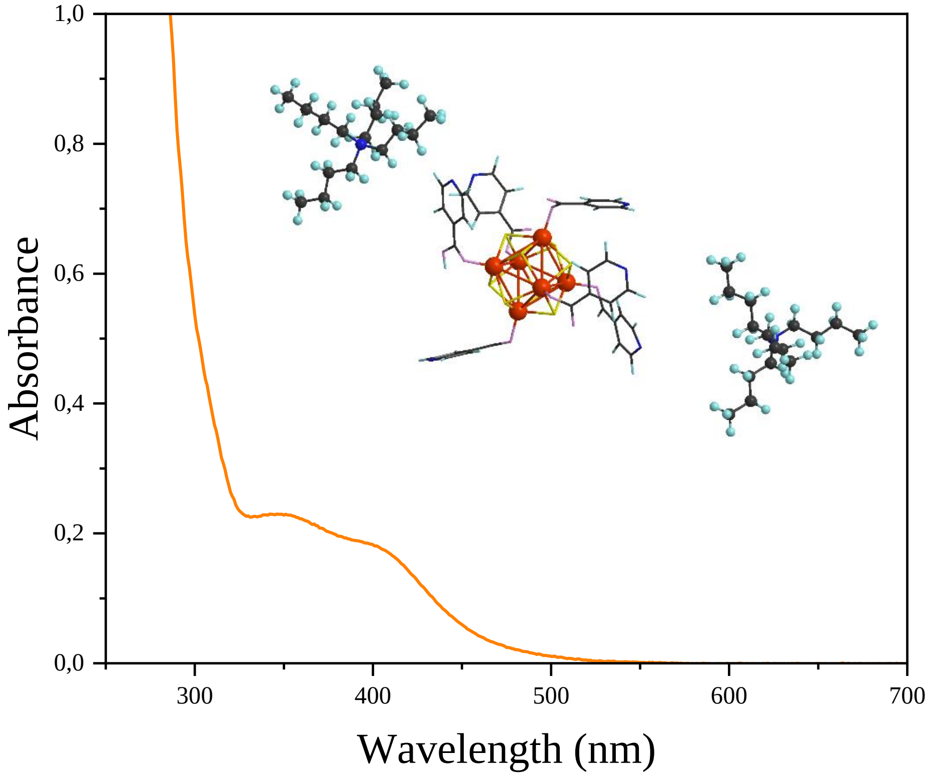

Figure S1. UV-Vis spectrum of Mo_6 in acetonitrile, showing the characteristic cluster absorption bands at 420 nm and 380 nm.

(a) (b)

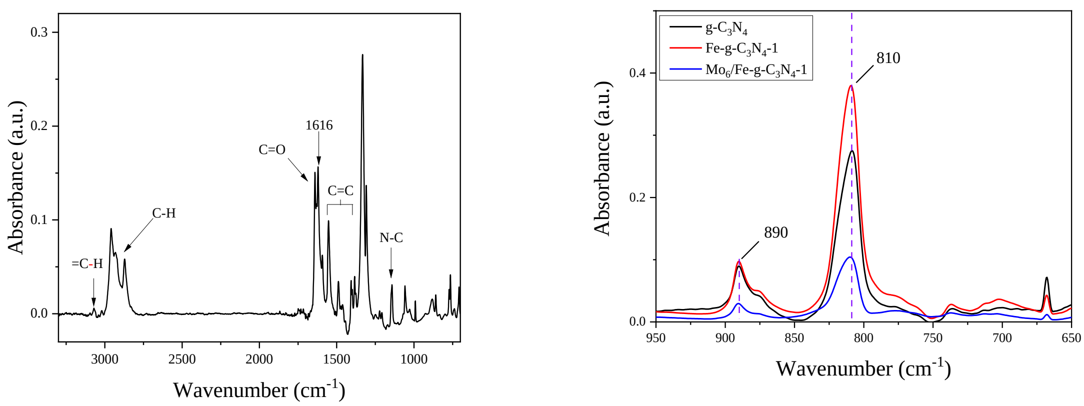

Figure S2. Fourier transform infrared spectra (FTIR) of (a) Mo_6 cluster, and (b) the approach of signal at 810 cm^{-1} of the hybrid materials. The FTIR spectrum of Mo_6 (a) displays several intense bands. The band located at 1642 cm^{-1} is due to the stretching of the carbon-carbon double bond, while the band at 3079 cm^{-1} is attributed to the stretching of the σ bond between sp^2-hybridized carbons and their attached hydrogens. The C–H stretching bands with sp^3 hybridization appear approximately between 2800–3000 cm^{-1}. The band at 1050 cm^{-1} corresponds to the N–C bond, as expected for tetrabutylammonium counterions. The four bands located at 1450, 1490, 1560, and 1590 cm^{-1} correspond to the aromatic C=C bonds of the isonicotinato ligands, while the band at 1616 cm^{-1} is attributed to the interaction of the hexametallic clusters with the carboxylate functionalities.

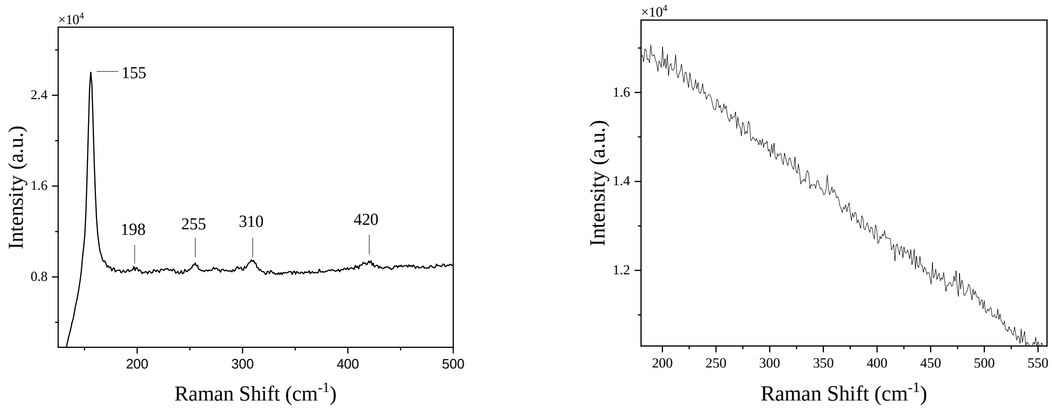

Figure S3. a) Raman spectrum of Mo_6 cluster. The signals identified at low Raman shifts are characteristic bands of the $\{Mo_6I_8\}^{4+}$ cluster core. (b) Raman region of the $Mo_6/Fe\text{-}g\text{-}C_3N_4\text{-}1$ sample measured. The spectra were registered at 785 nm.

(a) (b)

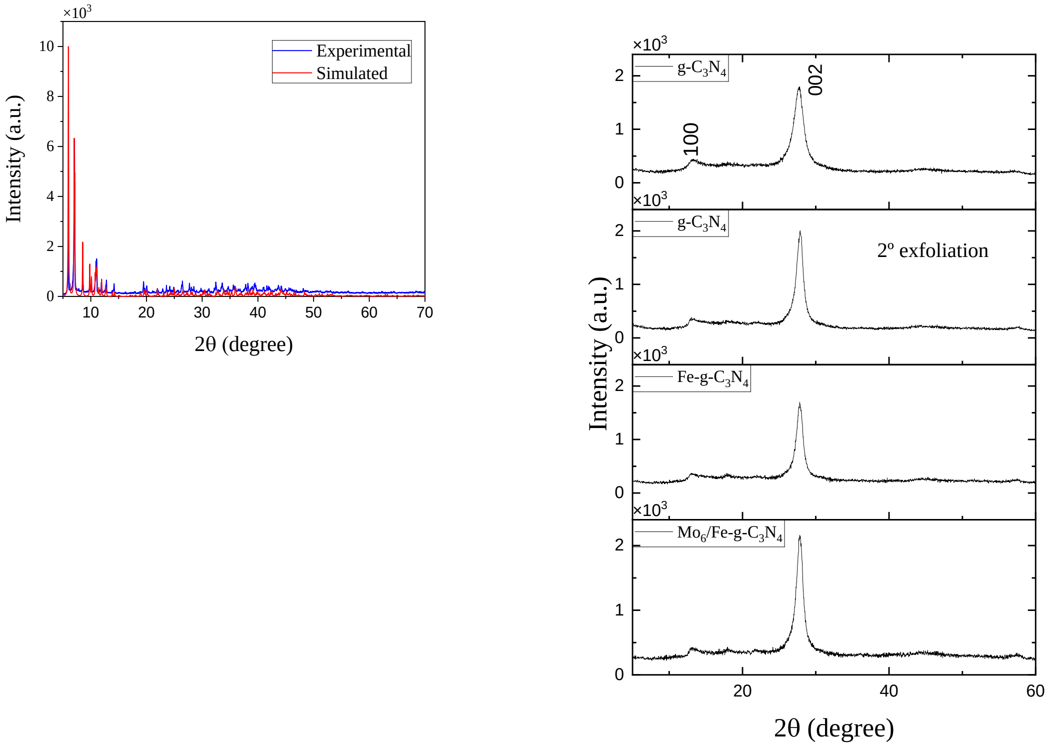

Figure S4. (a) Experimental and simulated PXRD patterns of Mo_6 compound; the matching of the diffraction peaks confirms the purity of the synthesized cluster [5]. (b) PXRD patterns of g-C_3N_4 based materials.

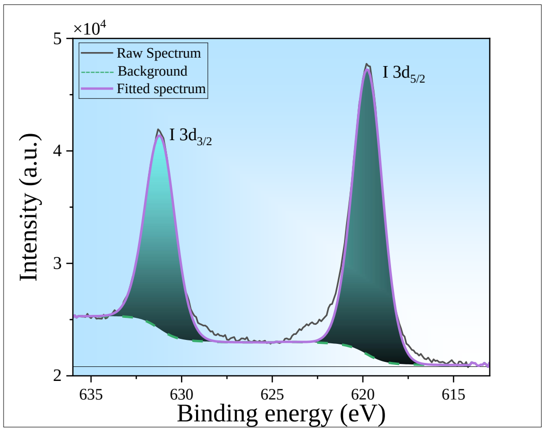

Figure S5. Iodine 3d region in XPS analysis of the $Mo_6/Fe\text{-}g\text{-}C_3N_4$ photocatalyst.

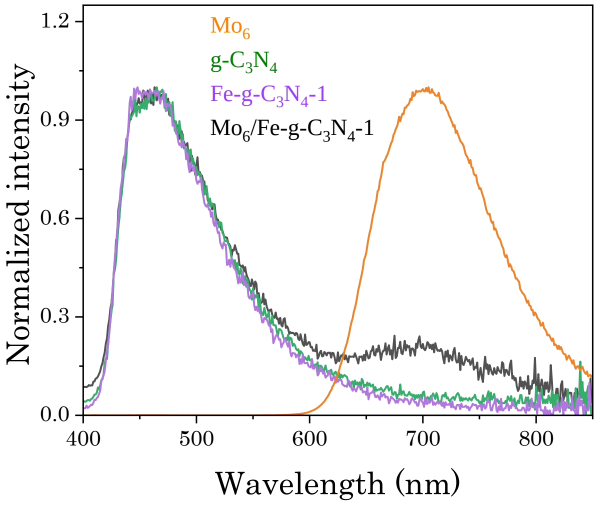

Figure S6. Steady-state emission spectra of Mo_6, $g\text{-}C_3N_4$, and the hybrid materials measured in acetonitrile under a N_2 atmosphere and $\lambda_{exc} = 365$ nm.

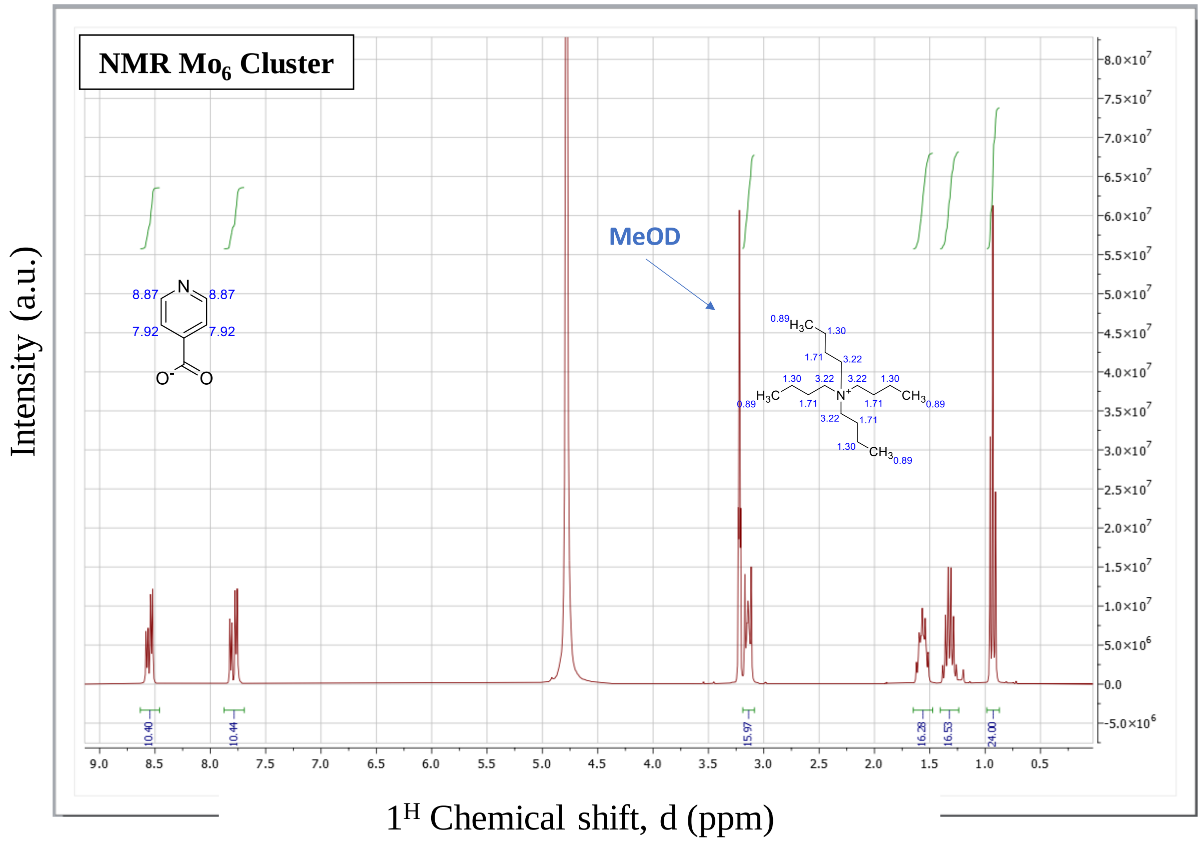

Figure S7. ^{1}H NMR spectra (MeOD, 25°C) of Mo_6. In the structures depicted in the figure, the theoretical values of chemical shift are included.

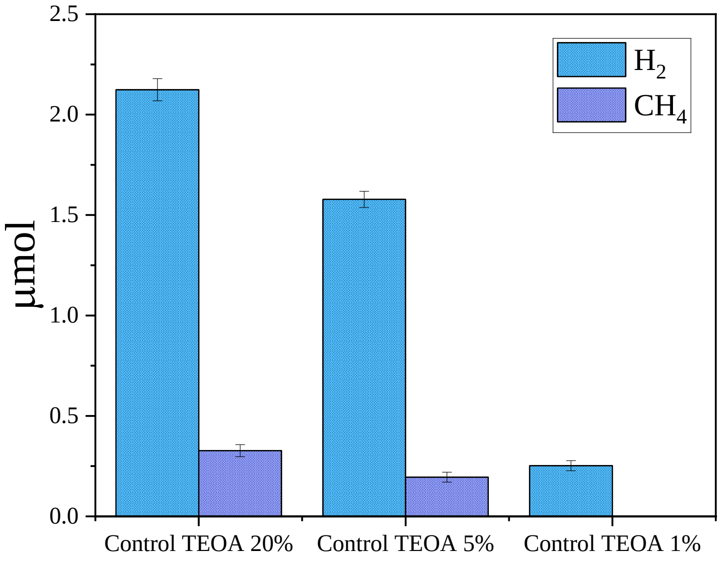

Figure S8. Gas production of photocatalytic control for optimization of the amount of sacrificial used in photochemical CO_2RR experiments. Only light and sacrificial agent (% in w/w) were employed in the solvent mixture. Figure S8 shows that after 24 h of reaction, the light degrades the TEOA; by a monoelectronic oxidation, a positively charged aminyl radical, who is a good oxidant, can be formed. This radical reacts with reduced species lead to produce a counterproductive back electron transfer. The reaction between aminyl radical and TEOA produce a carbon centered radical with an important reductive power ~1.0V [6]. As in the medium of control reactions there are only TEOA and acetonitrile, an auto-degradation was produced leading to the formation of CO_2, CH_4 and H_2 as final reaction products in the solutions with the highest amount of TEOA. In the case of the control reaction with TEOA 1% v/v, H_2 was detected.

Table S2. Control experiments for the CO_2RR studies.

**Test**	**Photocatalyst**	**Light**	**TEOA**	**Gas (µmol·g^{-1})**		
				**H_2**	**CH_4**	**CO**
**1**	Mo_6 cluster	Yes	Yes	384	164	3903
**2**	g-C_3N_4 pristine	Yes	Yes	55	3	0
**3**[a]	None	Yes	No	0	0	0
**4**[a]	None	Yes	Yes	1	0	0
**5**	Mo_6/Fe-g-C_3N_4-1	No	Yes	6	0	0
**6**	Mo_6/Fe-g-C_3N_4-1	Yes	No	0	0	0
**7**	Mo_6/Fe-g-C_3N_4-1	No	No	0	0	0

[a] The values of these measurements are expressed in µmol.

Table S3. Selection of g-C_3N_4 based materials in photocatalytic CO_2RR

**Sample**	**Light Source**	**Experimental conditions**	**Performance (μmol g^{-1} h^{-1})**	**Ref**
g-C_3N_4 (Ru(II) complex)	400W high-pressure Hg lamp, λ > 400 nm	4 mL of N,N-dimethylacetamide containing 20 % v/v of TEOA bubbled with CO_2	CO: 175; HCOOH: 150; H_2: 6.875	7
α-Fe_2O_3/g-C_3N_4	300W Xe lamp	CO_2 and water vapor	CO: 27.2	8
$FeWO_4$/g-C_3N_4	300W Xe lamp	20 mL of 0.5M Na_2SO_3	CO: 6	9
$SnFe_2O_4$/g-C_3N_4	300W Xe lamp	CO_2 and water vapor	CO: 7.56; O_2: 3.51	10
NH_2-MIL-101(Fe)/g-C_3N_4	300 W Xe lamp	CO_2, TEOA, CO_2 recirculation	CO: 22.14	11
Fe–g-C_3N_4	300 W Xe lamp	CO_2 and water	CO: 0.51	12
Fe_2O_3/g-C_3N_4	150 W Xe lamp	CO_2 and water	CH_4: 2.9×10^{-2}	13
Mo/g-C_3N_4	300 W Xe lamp	CO_2 and water	CO: 17.5; H_2: 37.3	14
g-C_3N_4/ZnO	300 W Xe lamp	CO_2 (generated *in situ* from $NaHCO_3$ and HCl) and water vapor	CH_3OH: 0.6	15
WO_3/g-C_3N_4 (Au-Ag cocatalysts)	diode (LED; Epitex, 30M32L), 435 nm, Intensity: 3.0 mW.cm^{-2}	CO_2 and water	CH_3OH: 0.1; HCOOH: 3.3×10^{-3}; CO: NR ; H_2: NR ; CH_4: NR.	16
g-C_3N_4/Bi_2WO_6	300 W Xe lamp	CO_2 and water vapor	CO: 5.19	17
Ti_3C_2/g-C_3N_4	300 W Xe lamp	CO_2 (generated *in situ* from $NaHCO_3$ and H_2SO_4) and water vapor	CO: 5.19; CH_4: 0.044	18
N-doped graphene/AgBr/ g-C_3N_4	UV cutoff filter $\lambda \geq 420$ nm; 150 mW cm^{-2}	Aqueous solution of $NaHCO_3$ and CO_2	CH_3OH: 21.18; C_2H_5OH: 51.29; CH_4: 18.04; CO: 0.18.	19
g-C_3N_4/Ti_3AlC_2	A 200 W Hg lamp; 150 mW cm^{-2}	H_2O/CH_3OH solution and CO_2	H_2: 13750; CO: 725; CH_4: 90.	20

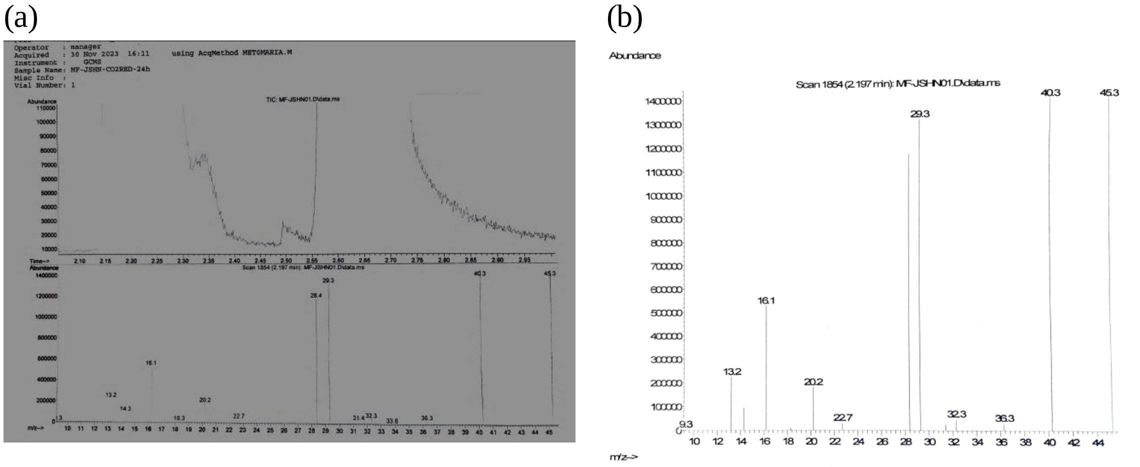

Figure S9. Carbon monoxide identification by GC-MS: (a) GC and (b) MS spectra.

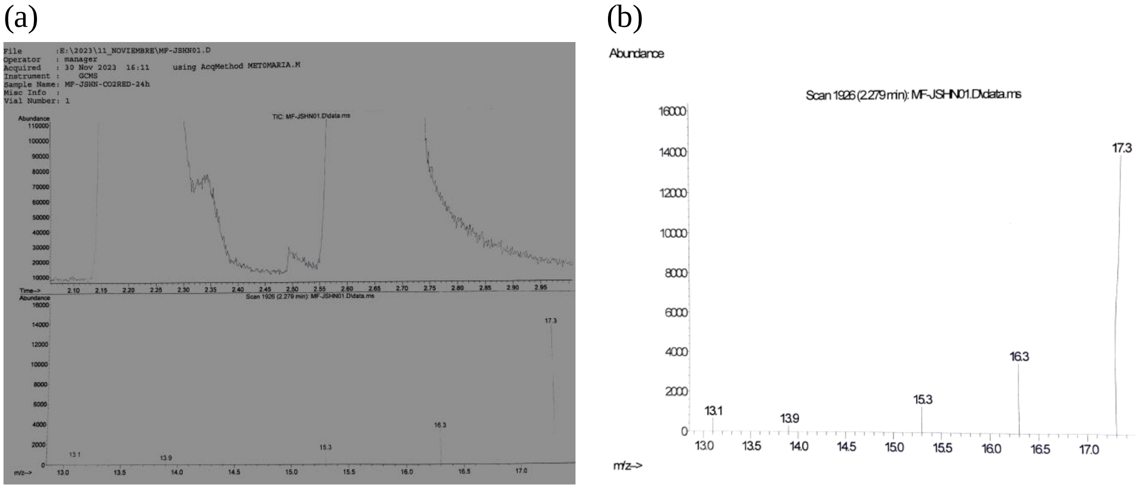

Figure S10. Methane identification by GC-MS: (a) GC and (b) MS spectra.

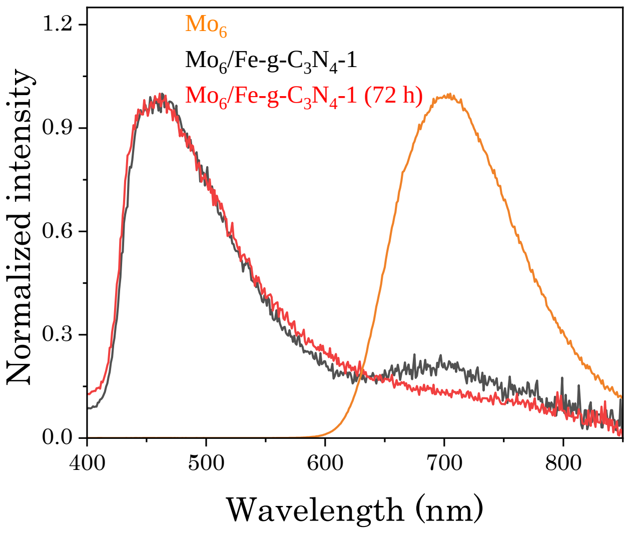

Figure S11. Photoluminescence spectra of the Mo_6/Fe-g-C_3N_4-1 material before and after 3 usage cycles (72 h), and of the Mo_6 compound.

3. REFERENCES

(1) Fuhrmann, A. D.; Pachel, F.; Ströbele, M.; Enseling, D.; Jüstel, T.; Meyer, H. J. Synthesis, Crystal Structure, and Luminescence of Metal Iodide Cluster Compounds $(NBu_4N)_2[M_6I_8(NCO)_6]$ with M = Mo, W. *Z Anorg Allg Chem* **2020**, *646* (19), 1650. https://doi.org/10.1002/zaac.202000209.

(2) Kirakci, K.; Cordier, S.; Perrin, C. Synthesis and Characterization of $Cs_2Mo_6X_{14}$ (X = Br or I) Hexamolybdenum Cluster Halides: Efficient Mo 6 Cluster Precursors for Solution Chemistry Syntheses. *Z Anorg Allg Chem* **2005**, *631* (2–3), 411. https://doi.org/10.1002/zaac.200400281.

(3) Todorova, N.; Papailias, I.; Giannakopoulou, T.; Ioannidis, N.; Boukos, N.; Dallas, P.; Edelmannová, M.; Reli, M.; Kočí, K.; Trapalis, C. Photocatalytic H_2 Evolution, CO_2 Reduction, and NOx Oxidation by Highly Exfoliated g-C_3N_4. *Catalysts* **2020**, *10* (10), 1. https://doi.org/10.3390/catal10101147.

(4) Van, M. N.; Mai, O. L. T.; Do, C. P.; Thi, H. L.; Manh, C. P.; Manh, H. N.; Thi, D. P.; Danh, B. Do. Fe-Doped g-C_3N_4: High-Performance Photocatalysts in Rhodamine b Decomposition. *Polymers (Basel)* **2020**, *12* (9), 1. https://doi.org/10.3390/polym12091963.

(5) Volostnykh, M. V.; Mikhaylov, M. A.; Sinelshchikova, A. A.; Kirakosyan, G. A.; Martynov, A. G.; Grigoriev, M. S.; Piryazev, D. A.; Tsivadze, A. Y.; Sokolov, M. N.; Gorbunova, Y. G. Hybrid Organic-Inorganic Supramolecular Systems Based on a Pyridine End-Decorated Molybdenum(II) Halide Cluster and Zinc(II) Porphyrinate. *Dalton Transactions* **2019**, *48* (5), 1835. https://doi.org/10.1039/c8dt04452j.

(6) Pellegrin, Y.; Odobel, F. Les Donneurs d'électron Sacrificiels Pour La Production de Combustible Solaire. *Comptes Rendus Chimie* **2017**, *20* (3), 283. https://doi.org/10.1016/j.crci.2015.11.026.

(7) Kuriki, R.; Ishitani, O.; Maeda, K. Unique Solvent Effects on Visible-Light CO_2 Reduction over Ruthenium (II)-Complex/Carbon Nitride Hybrid Photocatalysts. *ACS Appl Mater Interfaces* **2016**, *8* (9), 6011. https://doi.org/10.1021/acsami.5b11836.

(8) Jiang, Z.; Wan, W.; Li, H.; Yuan, S.; Zhao, H.; Wong, P. K. A Hierarchical Z-Scheme α-Fe_2O_3/g-C_3N_4 Hybrid for Enhanced Photocatalytic CO_2 Reduction. *Advanced Materials* **2018**, *30* (10), 1706108. https://doi.org/10.1002/adma.201706108.

(9) Bhosale, R.; Jain, S.; Vinod, C. P.; Kumar, S.; Ogale, S. Direct Z-Scheme g-C_3N_4/$FeWO_4$ Nanocomposite for Enhanced and Selective Photocatalytic CO_2 Reduction under Visible Light. *ACS Appl Mater Interfaces* **2019**, *11* (6), 6174. https://doi.org/10.1021/acsami.8b22434.

(10) Jia, Y.; Ma, H.; Zhang, W.; Zhu, G.; Yang, W.; Son, N.; Kang, M.; Liu, C. Z-Scheme $SnFe_2O_4$-Graphitic Carbon Nitride: Reusable, Magnetic Catalysts for Enhanced Photocatalytic CO_2 Reduction. *Chemical Engineering Journal* **2020**, *383*, 123172. https://doi.org/10.1016/j.cej.2019.123172.

(11) Dao, X. Y.; Xie, X. F.; Guo, J. H.; Zhang, X. Y.; Kang, Y. S.; Sun, W. Y. Boosting Photocatalytic CO_2 Reduction Efficiency by Heterostructures of NH_2-MIL-101(Fe)/g-C_3N_4. *ACS Appl Energy Mater* **2020**, *3* (4), 3946. https://doi.org/10.1021/acsaem.0c00352.

(12) Zhao, Z.; Liu, W.; Shi, Y.; Zhang, H.; Song, X.; Shang, W.; Hao, C. An Insight into the Reaction Mechanism of CO_2 Photoreduction Catalyzed by Atomically Dispersed Fe Atoms Supported on Graphitic Carbon Nitride. *Physical Chemistry Chemical Physics* **2021**, *23* (8), 4690. https://doi.org/10.1039/d0cp05570k.

(13) Nguyen, T. B.; Dinh Thi, T. H.; Pham Minh, D.; Bui Minh, H.; Nguyen Thi, N. Q.; Nguyen Dinh, B. Photoreduction of CO_2 to CH_4 over Efficient Z-Scheme γ -Fe_2O_3/g-C_3N_4 Composites. *J Anal Methods Chem* **2022**, *2022,* 1358437. https://doi.org/10.1155/2022/1358437.

(14) Zhang, R.; Li, P.; Wang, F.; Ye, L.; Gaur, A.; Huang, Z.; Zhao, Z.; Bai, Y.; Zhou, Y. Atomically Dispersed Mo Atoms on Amorphous G-C_3N_4 Promotes Visible-Light Absorption and Charge Carriers Transfer. *Appl Catal B* **2019**, *250*, 273. https://doi.org/10.1016/j.apcatb.2019.03.025.

(15) Yu, W.; Xu, D.; Peng, T. Enhanced Photocatalytic Activity of G-C_3N_4 for Selective CO_2 Reduction to CH_3OH via Facile Coupling of ZnO: A Direct Z-Scheme Mechanism. *J Mater Chem A Mater* **2015**, *3* (39), 19936. https://doi.org/10.1039/c5ta05503b.

(16) Ohno, T.; Murakami, N.; Koyanagi, T.; Yang, Y. Photocatalytic Reduction of CO_2 over a Hybrid Photocatalyst Composed of WO_3 and Graphitic Carbon Nitride (g-C_3N_4) under Visible Light. *Journal of CO_2 Utilization* **2014**, *6*, 17. https://doi.org/10.1016/j.jcou.2014.02.002.

(17) Li, M.; Zhang, L.; Fan, X.; Zhou, Y.; Wu, M.; Shi, J. Highly Selective CO_2 Photoreduction to CO over G-C_3N_4/Bi_2WO_6 Composites under Visible Light. *J Mater Chem A Mater* **2015**, *3* (9), 5189. https://doi.org/10.1039/c4ta06295g.

(18) Yang, C.; Tan, Q.; Li, Q.; Zhou, J.; Fan, J.; Li, B.; Sun, J.; Lv, K. 2D/2D Ti_3C_2 MXene/g-C_3N_4 Nanosheets Heterojunction for High Efficient CO_2 Reduction Photocatalyst: Dual Effects of Urea. *Appl Catal B* **2020**, *268*, 118738. https://doi.org/10.1016/j.apcatb.2020.118738.

(19) Li, H.; Gan, S.; Wang, H.; Han, D.; Niu, L. Intercorrelated Superhybrid of AgBr Supported on Graphitic-C_3N_4-Decorated Nitrogen-Doped Graphene: High Engineering Photocatalytic Activities for Water Purification and CO_2 Reduction. *Advanced Materials* **2015**, *27* (43), 6906. https://doi.org/10.1002/adma.201502755.

(20) Tahir, M. Construction of a Stable Two-Dimensional MAX Supported Protonated Graphitic Carbon Nitride (Pg-C_3N_4)/Ti_3AlC_3/TiO_3 Z-Scheme Multiheterojunction System for Efficient Photocatalytic CO_2 Reduction through Dry Reforming of Methanol. *Energy and Fuels* **2020**, *34* (3), 3540. https://doi.org/10.1021/acs.energyfuels.9b04393.
